# Democratising high performance computing for bioinformatics through serverless cloud computing: A case study on CRISPR-Cas9 guide RNA design with Crackling Cloud

**DOI:** 10.1371/journal.pcbi.1013819

**Published:** 2025-12-19

**Authors:** Jacob Bradford, Divya Joy, Mattias Winsen, Nicholas Meurant, Mackenzie Wilkins, Laurence O.W. Wilson, Denis C. Bauer, Dimitri Perrin

**Affiliations:** 1 School of Computer Science, Faculty of Science, Queensland University of Technology, Brisbane City, Queensland, Australia; 2 Centre for Data Science, Queensland University of Technology, Brisbane City, Queensland, Australia; 3 Australian e-Health Research Centre, Commonwealth Scientific and Industrial Research Organisation, Sydney, New South Wales, Australia; 4 Applied BioSciences, Faculty of Science and Engineering, Macquarie University, Macquarie Park, New South Wales, Australia; 5 Australian e-Health Research Centre, Commonwealth Scientific and Industrial Research Organisation, Adelaide, South Australia, Australia; 6 School of Medical Sciences, Department of Biomedical Informatics and Digital Health, University of Sydney, Sydney, New South Wales, Australia; Burnet Institute, AUSTRALIA

## Abstract

Organisations are challenged when meeting the computational requirements of large-scale bioinformatics analyses using their own resources. Cloud computing has democratised large-scale resources, and to reduce the barriers of working with large-scale compute, leading cloud vendors offer serverless computing, a low-maintenance and low-cost model that provides ample resources for highly scalable software applications. While serverless computing has broad use, its adoption in bioinformatics remains poor. Here, we demonstrate the most extensive use of high-performance serverless computing for bioinformatics by applying the available technologies to CRISPR-Cas9 guide RNA (gRNA) design. Our adaptation of the established gRNA design tool, named Crackling, implements a novel, cloud-native and serverless-based, high-performance computing environment using technologies made available by Amazon Web Services (AWS). The architecture, compatible with technologies from all leading cloud vendors, and the AWS implementation, contributes to an effort of reducing the barrier to large computational capacity in bioinformatics and for CRISPR-Cas9 gRNA design. Crackling Cloud can be deployed to any AWS account, and is freely available on GitHub under the BSD 3-clause license: https://github.com/bmds-lab/Crackling-AWS

## Author summary

Cloud computing platforms have changed how researchers access and use computing resources, yet its full potential in bioinformatics remains largely unrealised. To help overcome the barriers researchers face when needing high-performance computing, we propose using publicly available cloud platforms to run bioinformatics tools. We focus on serverless systems that operate only when needed, scale automatically, and reduce costs. This is an ideal combination for researchers who run large-scale analyses infrequently. In this work, we developed Crackling Cloud as a serverless system for gene editing research. It includes both a reusable cloud architecture and a working implementation that can be deployed to any Amazon Web Services account, allowing researchers to run experiments without specialised hardware or technical expertise. We tested Crackling Cloud on datasets of varying size and showed that it performs well as the complexity of the task increases. The system is open-source, making it easy to adopt and share. We believe this approach can help democratise access to bioinformatics tools and promote broader use of cloud technologies in scientific research.

## Introduction

Cloud computing has revolutionised large-scale and high-performance computing, offering unprecedented flexibility and cost-efficiency. These advancements can benefit computational biology and bioinformatics, as they face an increasing growth of data and the complexity of its analysis [1,2]. While there are many examples of traditional server-based environments being used for bioinformatics pipelines, there is limited use of serverless-based technologies.

### Modern cloud computing for bioinformatics

Traditional server-based architectures are useful for large and long-running computational tasks, however, they are only economically sound when their average utilisation is kept to a maximum. Furthermore, servers require routine maintenance in terms of software updates, security patching, and replacement of hardware, and developers must be well-versed in software engineering practices to benefit from the resources available. In the research context, these conditions are difficult to satisfy, and servers can sit idle for long periods, leading to waste and high costs. The alternative model of serverless computing can be much more efficient. Application code is provided by the developer for it to be executed in a pre-defined server environment managed by the cloud vendor and only when selected events occur. That could be when a file is uploaded or a database record created. As the frequency of events increases, serverless platforms automatically scale by invoking additional execution environments, supporting thousands of parallel functions by default without manual provisioning. This model improves the pace at which software can be developed as server maintenance is only a concern to the vendor and not the developer, and keeps costs low, as the infrastructure can scale down to zero when there is no work to complete, unlike traditional server-based architectures that remain running.

In bioinformatics, the viability of serverless technologies has been demonstrated for large-scale analyses but they remain less used compared to other high-performance computing environments [3,4].

To date, there have been only eight demonstrations: a pipeline for base-calling Nanopore reads [5], two variant-calling pipelines [6,7], a sequence comparison tool [8], a two-dimensional DNA visualisation web service [9], a web tool for genetic manipulation of microorganisms [10], and two RNA-seq workflows [11,12].

These have demonstrated that serverless environments can greatly benefit bioinformatics through simplifying the software development effort, significantly reducing run-time compared to their server-based counterparts, while achieving this with reasonable cost. However, existing implementations have not provided a reusable cloud architecture template for others to build upon, which also prevents the software from being deployable to other cloud accounts. Given the increasing volume of omics data and the demand for larger computational capacity, serverless cloud computing presents a significant opportunity in bioinformatics research, including the computationally challenging task of designing high-quality CRISPR-Cas9 guide RNA (gRNA).

### High-performance computing for CRISPR technologies

CRISPR-Cas9 has become the gold-standard technology for gene editing due to its simplicity and low cost. When a CRISPR-Cas9 nuclease is provided with a gRNA, it can introduce a double-stranded DNA break at nearly any genomic loci of interest, giving the opportunity for that DNA site to be edited with great precision [13]. It has truly modernised the gene editing toolset and has enabled large-scale and robust genomic studies.

When choosing a good quality gRNA, two critical and computationally demanding properties must be evaluated: (1) the on-target activity and (2) the off-target activity. A good quality gRNA is one that has maximal on-target activity and minimal off-target activity.

The complexity of gRNA design depends on the specific genome being targeted. The quality of a gRNA in one genome does not necessarily translate to another. As a result, gRNA quality must be evaluated for each genome of interest, and therefore, gRNA design tools must scale to genomes of any size.

In our previous benchmarking study, we found that standalone gRNA design tools, when run on high-performance computers, often failed to scale to large genomes due to resource saturation or they were exceedingly slow [14]. The most basic implementations used single-threaded code designed for a single CPU core. Some implementations used multi-threaded code to use all cores of the CPU, and some others explored the use of hardware accelerators like GPUs and FPGAs [15].

While that study explored tools that can be ran on local workstations, here, our focus is on those available online as they are more likely to utilise public cloud technologies. We have investigated the number of tools listed in two community databases that can process any specified or provided genome.

In the first database, 49 of the 69 listed methods are available as web servers but 20 of those were offline [16]. Among the available web servers, three allow the user to specify the genome to analyse, 18 have a limited list of pre-selected genomes, and eight do not consider a genome-wide screen at all. In the second database, 77 of 105 listed methods are available as web servers and only six of those allow the user to provide the genome [17]. Among these, two were offline, two were not gRNA design methods and one was a commercial tool claiming 124,000 genomes in their database. The remaining tool was already listed in the first database. The full lists are available in S1 Table and S2 Table.

Of the tools that allow users to specify a genome, the ‘Eukaryotic Pathogen gRNA Design Tool’ (EuPaGDT) limits genome size to less than 200 megabytes [18], Benchling can analyse any chosen genome but is a commercial product, and CRISPy-Web allows the user to upload a custom genome [19] without a size limit.

CRISPy-Web is one of few online tools that implements a scalable cloud architecture [19]. It uses multiple, decoupled service workers that are tied together by a message queue, enabling it to scale to large data. The web interface communicates with the back-end via a Representational State Transfer (REST) interface. Having that REST interface can allow end-users to implement their own tools to interact with the service. However, upon inspecting the available source code of CRISPy-Web, we could not find documentation to support deploying it into another cloud account.

While offline tools can be run using server-based cloud infrastructure, they would still be constrained by the limited resources of a single server.

Currently, no gRNA design tool implements a scalable, serverless architecture.

### Serverless computing for CRISPR technologies

The evaluation of gRNA quality is a parallelisable problem. The analysis of on-target and off-target activity of each gRNA can co-occur. Our previously published method for designing good quality gRNA, named Crackling, is amongst the fastest tools available. Crackling takes advantage of multi-threading on the CPU and bit-wise operations, but not any acceleration technologies, nor any scalable cloud technologies.

To select gRNA based on on-target activity, Crackling evaluates each gRNA using up to three independent methods [20]. That approach is more precise than any individual method alone and has resulted in our experiments seeing successful edits up to 99% of the time [21]. However, that means multiple methods must be executed rather than just one.

Evaluating gRNA off-target activity is the most time-consuming step in the design process. Toward overcoming that, Crackling extracts closely related CRISPR sites using a specialist index.

While Crackling is fast and accurate, not always do end-users have the necessary resources to run the program, and they may not necessarily have the expertise to work with a bioinformatics program. That is true for all gRNA design methods, as they need to analyse entire genome sequences and are usually available as source code. Therefore, users often rely on the tools that are available on the internet or lend efforts to employing a traditional high-performance computing environment.

In an effort to overcome barriers to high-performance computing environments and the challenges of working with offline tools, we are turning to the public cloud so users can deploy, from a template, a gRNA design protocol to a democratic computing platform. No other gRNA design tool has done that. Specifically, we are leveraging the dynamic compute environments made available by Amazon Web Services (AWS).

Crackling Cloud is the first free, serverless-based CRISPR gRNA design pipeline, and is publicly available on GitHub as open-source software under the terms of the BSD 3-clause licence: https://github.com/bmds-lab/Crackling-AWS

## Design and implementation

### Software architecture

**Fig 1:**
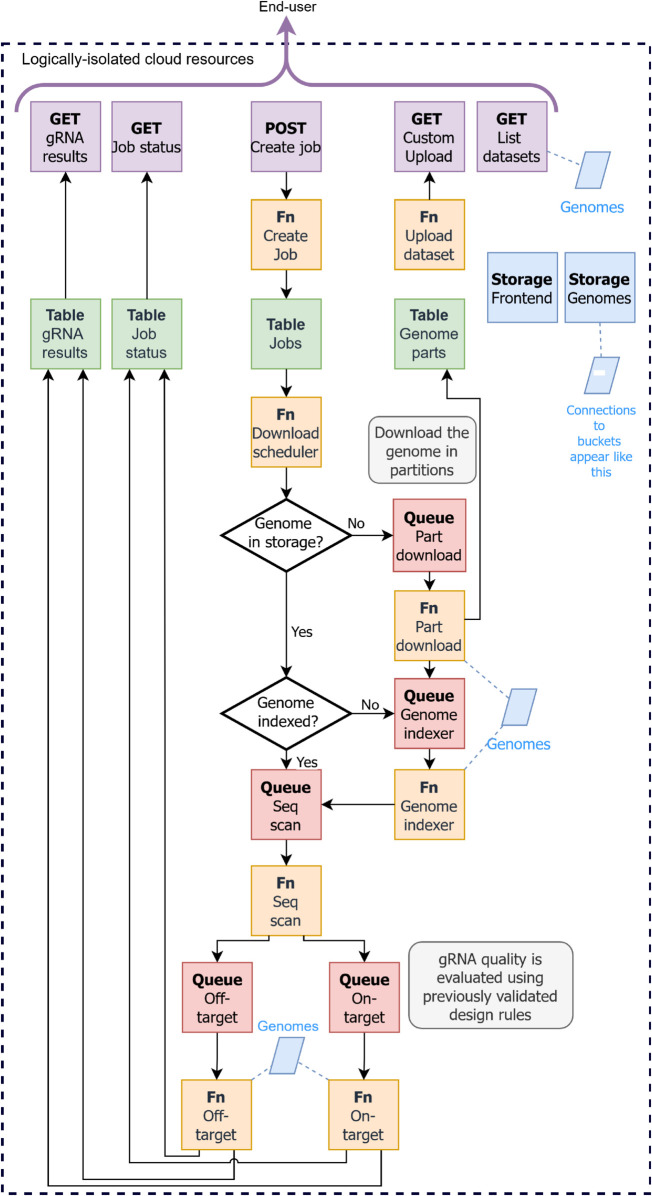
Cloud architecture diagram of Crackling Cloud. Each colour represents a cloud service. Lavender indicates API endpoints, orange indicates function-based compute, green indicates database tables, blue indicates persistent file storage, and magenta indicates message queues. Arrows between blocks show data flow. Each deployment of the software operates within a designated cloud account. Any good cloud vendor is certified to provide logically isolated resources from one customer to the next. The gRNA service is accessible via an HTTP API or the accompanying web client.

Crackling Cloud is an event-driven pipeline that leverages a flexible, serverless architecture that could be implemented using the technologies of most modern cloud vendors. We selected Amazon Web Services (AWS) based on their position as the leading vendor and their robust service offering. AWS provides cloud services to many of the largest companies in the world, and to many research and education institutions. This section describes the implementation of Crackling Cloud. See Fig 1 for a vendor-agnostic architecture diagram.

The entry point to the pipeline is via a HTTP API served through Amazon API Gateway. There are two critical endpoints that enable an end-user to design gRNA: submit job and retrieve results. To submit a job, the end-user provides the sequence of the gene to target, and to measure gRNA specificity across the entire genome, they provide a National Center for Biotechnology Information (NCBI) Genome identifier. In return, a job identifier (ID) is provided. Upon submitting a job, the analysis infrastructure spins up from zero. The volume of needed resources is determined by the number of gRNA to assess. Jobs are submitted in series but run in parallel. gRNA are also assessed in parallel.

If users want to design gRNA for a genome not available in the NCBI Genome database, they can securely upload their own genome to a private Amazon Simple Storage Service (S3) bucket. S3 functions as an object storage service similar to a conventional file system. The uploaded genome will then be listed alongside the option to provide a genome accession.

Results are associated with the job ID and can be retrieved by querying the second API endpoint or through a provided web-based interface. Alternatively, the HTTP API enables an advanced user to retrieve results using their own method (e.g., a custom-built software script, their own graphical interface, Excel, etc.).

Upon a job being submitted via a HTTP request, a database record is created in Amazon DynamoDB. The event of creating that record triggers further data preparation steps, orchestrated by AWS Lambda and Amazon Simple Queue Service (SQS).

Lambda is a serverless run-time environment: no provisioning of servers is required; AWS handles that in the background as needed. Only the code and any dependencies not installed by AWS are provided by the developer. SQS is a queuing service that triggers Lambda to automatically handle, process and scale tasks based on the queued workload. Lambda can receive messages (tasks) from a SQS queue in batches. Each Lambda invocation processes batches of messages for up to 15 minutes, and without exceeding the 10 gigabyte memory limit. By default, an AWS account can invoke up to 1000 concurrent Lambda run-time environments. This exceeds the number of concurrent threads that any local workstation could execute. We have configured the size of batching for each SQS-to-Lambda integration based on the specific requirements of the task. When the queue exceeds the current processing capacity, Lambda automatically handles increasing concurrency to handle the workload.

Once a job is submitted to the database, a Lambda function checks that the specified genome is available in storage. If it is not, the NCBI Genome database is queried using the provided accession to retrieve metadata, including the size of the genome. For genomes larger than 50 megabytes, the download is divided into portions, with each portion queued as a byte range of the original file. This approach allows the genome download to be parallelised across multiple Lambda invocations, improving efficiency and avoiding the 15 minute time-limit of Lambda. A subsequent Lambda function handles downloading each portion to S3. After all portions have been obtained, S3 merges the portions.

After merging, an index of all CRISPR-targetable sites is generated. This is critical to assess the off-target risk of candidate gRNA. To index the sites, a specialist data structure, named Inverted Signature Slice Lists, is used, as implemented in the standalone edition of Crackling [22]. The task of generating the ISSL index is queued and handled by a specialist Lambda function.

Upon the index becoming available, a subsequent Lambda function processes the provided gene sequence. Candidate gRNA are extracted using a regular expression pattern that matches the conventional SpCas9 Protospacer-Adjacent Motif (i.e., 21 nucleotides followed by GG, and the complement for the reverse strand). Each candidate gRNA is added to two separate queues: one for assessing on-target activity and another for assessing off-target activity. The tasks in those queues are consumed by Lambda functions respective to the queue’s purpose.

Due to the asynchronous design, a third API endpoint is available to obtain a progress update. Upon the assessment of each gRNA property, results are written to the database and can be retrieved via the API or the provided web client.

This architecture is designed to dynamically allocate resources based on workload, allowing for parallel processing of gRNA assessments. On a typical workstation, similar tasks involving genome-wide off-target analysis can take several hours or days, especially for large genomes [14]. In contrast, Crackling Cloud leverages serverless computing platforms to distribute tasks across hundreds of serverless computing environments. This elasticity ensures that the infrastructure scales up during analysis to reduce run-time and scales down to zero when idle, resulting in cost-efficiency that is difficult to achieve with traditional server-based setups. Furthermore, the pay-per-use model of serverless computing eliminates the need for maintaining idle resources, making it particularly attractive for infrequent yet large workloads common in bioinformatics research.

## Results

### Performance benchmark

We previously benchmarked the performance of CRISPR-Cas9 gRNA design tools, in terms of speed and accuracy [14,22]. Crackling was amongst the fastest and most accurate tools available. Here, we have benchmarked Crackling Cloud in terms of speed, granted that the gRNA selection process remains the same as the standalone tool, and therefore, its accuracy does not change. The same genome assemblies have been used.

Run-time was measured from when the job was submitted via the API to the completed assessment of every gRNA. That included the time to download and process the genome, which is unlike the previous benchmark, that only included gRNA assessment and excluded time spent downloading and indexing the genome. Our intent was to measure the actual time that an end-user would wait for all results to become available.

Each experiment was performed as a *warm* run, meaning the infrastructure had already been initialised and was standing by, ready for processing, before timing out and scaling back to zero. In contrast, a *cold* run would have involved additional overhead from launching the serverless architecture, resulting in longer execution times—a typical characteristic and side effect of the serverless cloud computing model. The duration of a cold start can vary from a few milliseconds to over a second.

To measure the impact of genome size, we analysed genomes of varying size from the NCBI Genome database. The sequence of the *rrs* gene (16S ribosomal RNA; NCBI gene ID 2700429) was used for each test. rrs was selected as representation of any real sequence that may be provided. It contains 260 candidate gRNA.

To measure the impact of the number of gRNA to assess, we generated artificial gene sequences containing a precise number of gRNA. We used these gene sequences and the O. sativa genome (GCF_001433935.1) as inputs.

Analysis times were measured for Crackling Cloud under two conditions: varying genome size and varying gRNA count. This allowed us to quantify how the architecture scales with input complexity.

Although the standalone version of Crackling previously processed 10,000 guides in under a minute [23], the benchmarks presented here are not directly comparable. In this study, run-time measurements include the time required to download and pre-process the genome, whereas earlier benchmarks considered only gRNA quality assessment. Both approaches exclude installation or deployment time. Furthermore, Crackling Cloud introduces a fundamentally different approach, leveraging an event-driven, serverless architecture rather than traditional server-based systems. This architectural shift makes directly comparing run-time less meaningful, even though maintaining low execution time remains an important expectation for end-users.

### Increasing genome size

When the rrs gene was provided as input for all tests but the genome selected for off-target assessment varied, the analysis time increased proportionally by the number of CRISPR target sites in the associated genome. Importantly, genome size does not simply impact the number of CRISPR sites; rather, GC content and repetition have a more significant impact. Although the total time for Crackling Cloud to download and process a genome is longer than the reported gRNA assessment time of standalone Crackling, it remains within a practical range for completing a bioinformatics analysis. See Table 1 for results.

**Table 1:** Time taken to process the rrs gene sequence with an increasing genome size.

Genome (Accession)	Size (MB)	Number of CRISPR sites	Run-time (seconds)
*Oryza sativa* (GCF_001433935.1)	374.4	61.7*10^6^	420
*Panicum hallii* (GCF_002211085.1)	507.4	91.9*10^6^	667
*Xiphophorus couchianus* (GCF_001444195.1)	685.5	100.6*10^6^	694

### Increasing number of gRNA to assess

When the O. sativa genome was selected as a reference for off-target scoring, and the number of gRNA in an artificial gene sequence was increased from 1000 to 10,000, the analysis time increased proportionally with the number of gRNA. Crackling Cloud analysed 1000 gRNA in 69 seconds, and 10,000 gRNA within 3.5 minutes. See Table 2 for all results.

**Table 2:** Time taken to process the O. sativa genome with an increasing the number of gRNA to assess.

Number of gRNA	Run-time (seconds)
1000	69
2000	84
3000	102
4000	117
5000	152
6000	176
7000	170
8000	178
9000	195
10000	209

### Cost analysis

The cost of running the software on AWS was measured using genomes of increasing size, all targeting the same *rrs* gene as in previous experiments. See Table 1 for the list of genomes. Each experiment executed in a separate deployment, allowing precise measurement of the cost per run. All experiments were run within the same hour in September 2025 to ensure consistent and comparable cost measurements. Designing 260 guides for O. sativa incurred a cost $0.25 USD, P. hallii $0.31 USD and X. couchianus $0.43 USD. These figures reflect the total AWS resource usage and excluded any free-tier benefits. An on-going cost of $0.025 USD per hour was associated with the virtual network that operated while the software was deployed. After destroying the cloud infrastructure and data in storage, no further costs were incurred.

As demonstrated by these measured cloud computing costs, Crackling Cloud is less expensive to deploy and run than having a dedicated local hardware environment, which reflects a key advantage of public cloud computing. Local setups require an initial capital investment, and their computing resources are fixed, creating scalability constraints unless additional upgrades are purchased. In contrast, cloud platforms elastically allocate resources, enabling workloads to scale dynamically. This elasticity is supported by the vendor’s large-scale investment in distributed data centers, which provide virtually unlimited computational capacity for typical bioinformatics workloads. Combined with elastic scalability, the low operational cost positions Crackling Cloud as a cost-effective alternative to procuring and maintaining dedicated local hardware.

## Availability and future directions

Crackling Cloud is available under the terms of the BSD 3-clause licence and can be accessed via GitHub at https://github.com/bmds-lab/Crackling-AWS. Issues or unexpected behaviour can be reported to us via the GitHub repository. We will investigate these as soon as possible and provide assistance as necessary. Contributions from the community are welcome and can be submitted to us via a GitHub Pull Request.

The software is built using the AWS Cloud Development Kit (CDK), which enables infrastructure-as-code using popular programming languages. We used the Python interface, following object-oriented programming principles. The CDK simplified software development efforts, and increased the robustness and security of the software. As a result, Crackling Cloud is available as a reusable, cloud-native solution for any AWS account, and can be extended by anyone with skills in object-oriented programming. The architecture is modular and extensible, enabling other bioinformatics methods to be substituted into the compute components. For example, a serverless-based read aligner could be built by accepting FASTQ files instead of gene sequences, and using the NCBI genome accession to build a Bowtie index. Downstream components could then be modified to perform alignment rather than gRNA assessment.

Although Crackling Cloud uses a different approach to traditional server-based systems, our results show clear advantages of event-driven and serverless designs. Bioinformatics analyses finish in minutes, costs can be as low as $0.25 per run, and the system scales automatically without user input. Standalone machines are restricted by fixed hardware and need ongoing maintenance, while elastic cloud capacity supports thousands of parallel executions and handles large datasets that would overwhelm local resources. Users also avoid upfront hardware costs, benefiting instead from a low-cost pay-per-use model. These features make cloud an appealing option for researchers needing accessible, high-performance bioinformatics tools.

Importantly, bioinformatics methods intended for this architecture must be designed with the constraints of a serverless environment in mind: for AWS Lambda, that is, a maximum execution time of 15 minutes and a memory limit of 10 gigabytes. In addition to these resource limits, serverless functions must follow a stateless design pattern, meaning they cannot retain data or context between invocations. Any required state must be passed as input or retrieved from external storage services such as object stores or databases. If a method cannot be adapted to operate within these constraints, due to time or memory requirements, or reliance on persistent state, using a traditional virtual machine may be more appropriate. Documentation is provided to support future development efforts, including instructions for making changes.

As demonstrated here, cloud computing platforms are not limited to hosting web services; they offer flexible and powerful computational infrastructure suitable for scientific workflows. Our key contribution is the underlying cloud-native architecture, which can be adapted for other bioinformatics tools. We propose that future work consider adopting a serverless architecture for portability, accessibility, scalability and cost-efficiency.

## Supporting information

S1 Table List of reviewed software from Awesome-CRISPR. (XLSX)

S2 Table List of reviewed software from WeReview. (XLSX)
